# Impact of histological and clinical parameters on resection status and recurrence probability in head and neck basal cell carcinomas

**DOI:** 10.3389/fonc.2025.1679804

**Published:** 2025-11-24

**Authors:** Felix Deffner, Givi Magradze, Kia Melzer, Anna Charlotta Schlieper, Andreas Knopf, Naglaa Mansour, Christoph Becker, Manuel Christoph Ketterer

**Affiliations:** Department of Oto-Rhino-Laryngology, Medical Center – University of Freiburg, Freiburg, Germany

**Keywords:** basal cell carcinoma, histology, resection, recurrence, surgical management

## Abstract

**Objectives:**

This study aims to identify histological and clinical parameters associated with an R1 resection status and the recurrence rate in surgically treated basal cell carcinomas (BCC) and to evaluate the impact of an initial R1 resection status on the likelihood of tumor recurrence. Understanding these associations is essential for optimizing surgical treatment strategies and reducing the risk of BCC recurrences.

**Methods:**

This retrospective single-center study analyzed primary head and neck BCC surgically treated between 2019 and 2024 to evaluate patient and tumor characteristics, including histological subtype, tumor location, resection status, and recurrence rate. R0 was defined as complete excision with a safety margin of at least 3 mm or histopathological confirmation of complete tumor removal with tumor-free resection margins.

**Results:**

Among 241 cases of head and neck BCC, an initial R1 resection status was significantly associated with the sclerodermiform subtype and auricular localization. Tumor clearance was achieved within one or two surgical stages in approximately 80% of cases. Despite these risk factors, organ preservation was possible in over 93%, and local anesthesia proved sufficient in 90% of procedures. The recurrence rate remained low at 2.1%. Reconstructive techniques were frequently required, with local flaps and skin grafts being the most used methods.

**Conclusion:**

This study highlights the effectiveness of outpatient procedures under local anesthesia on the one hand and the tissue-sparing and organ-preserving approach on the other for head and neck BCC, achieving high R0 resection and organ preservation rates. Incomplete resection was linked to the sclerodermiform subtype and auricular location. Despite these risks, recurrence was rare. The frequent use of reconstructive techniques reflects the focus on aesthetic and functional outcomes.

## Introduction

Basal cell carcinoma (BCC) is the most common malignant skin tumor worldwide, accounting for approximately 80% of all non-melanoma skin cancers (NMSC). The incidence of BCC has been rising, particularly among individuals with fair skin and significant cumulative sun exposure (1). The primary risk factor for BCC development is ultraviolet radiation (UV), which induces DNA damage and mutations in key regulatory genes. Additional risk factors include immunosuppression, genetic predisposition (e.g., Gorlin syndrome), and exposure to ionizing radiation or certain carcinogenic chemicals (2).

BCCs predominantly occur in sun-exposed areas, with the head and neck being the most commonly affected regions due to chronic UV exposure. Clinically, BCCs present as slow growing, locally invasive tumors with very low metastatic potential. However, their invasive behavior can cause significant morbidity, especially when lesions develop in cosmetically or functionally critical areas such as the periorbital region, nose, or ears (3). BCCs exhibit various histological subtypes, which are recognized in current clinical guidelines. For example, the German S2k guideline (01/24) recommends that, when applicable, the histological subtype should be clearly specified in the pathology report (4). This distinction is important, as certain aggressive subtypes—such as infiltrative, morpheaform, and basosquamous variants—tend to invade deeper tissues and pose significant therapeutic challenges despite the generally indolent course of BCCs (5).

The standard treatment for BCC is surgical excision, with histopathological margin assessment ensuring complete tumor removal while preserving as much healthy tissue as possible (6). In cases where surgery is contraindicated or not feasible, non-surgical modalities such as radiotherapy, topical therapies (e.g., imiquimod, 5-fluorouracil), or targeted systemic treatments (e.g., Hedgehog pathway inhibitors such as vismodegib and sonidegib) may be considered (7). In select cases, Mohs micrographic surgery (MMS) represents a valuable surgical approach, particularly for high-risk tumors located in cosmetically or functionally sensitive areas. MMS involves the stepwise excision of the tumor with immediate microscopic evaluation of the entire surgical margin, enabling maximal tissue preservation while ensuring complete tumor clearance (8).

Despite the generally favorable prognosis of BCC, achieving complete tumor clearance during surgical excision remains a key challenge. An initial R1 resection status, characterized by microscopic tumor involvement at the surgical margins, is associated with a significantly increased risk of local recurrence (9). However, the factors contributing to an R1 status are not yet fully understood and may be influenced by specific histopathological features of the tumor.

Having this background in mind, this study aims to address the following key questions:

What histological and clinical parameters are associated with an R1 resection status and recurrence rate in BCC? How does an R1 resection status influence the likelihood of tumor recurrence? Answering these questions is crucial for optimizing treatment strategies in the challenging balance between achieving functionally and aesthetically satisfactory outcomes and maintaining oncological safety, particularly in high-risk patients requiring complex reconstructions.

## Materials and methods

### Study design

This retrospective study analyzed cases of primary surgical treated BCC located in the head and neck region. All patients were treated at the Department of Oto-Rhino-Laryngology, Medical Center and Faculty of Medicine, University of Freiburg, between January 2019 and December 2024. The study was approved by the local ethics committee (approval number: 24-1493-S1-retro) and is registered in the Freiburg Clinical Trial Register (FRKS; registration number: 006002) and the German Clinical Trial Register (DRKS; registration number: DRKS00037588). All data were pseudonymized prior to analysis in accordance with institutional ethical standards.

The principal objective of this study was to assess the impact of selected histopathological and clinical parameters on the likelihood of incomplete tumor resection, indicated by an R1 resection margin, as well as on tumor recurrence.

Histopathological parameters included histological subtype, tumor localization, depth, and width. Clinical parameters encompassed age at initial diagnosis, sex, type of anesthesia, organ preservation and use of reconstructive techniques.

Eligibility criteria included a confirmed histopathological diagnosis of BCC located within the head and neck region. Only patients aged 18 years or older who underwent initial curative-intent surgical treatment at our institution were included. Patients were excluded if they were under the age of 18, had incomplete medical records, had undergone prior surgical treatment for the tumor at an external facility or were treated non-surgical.

R0 was defined as complete excision with a safety margin of at least 3 mm or histopathological confirmation of complete tumor removal with tumor-free resection margins. Patients who underwent a simple biopsy were classified as R1, as this procedure is performed solely to determine the tumor entity and does not aim for R0 resection. Biopsies and excisional biopsies were analyzed together, since only procedures performed as part of the initial step of a planned in sano (R0) resection were included in the analysis.

### Statistical analysis

Patient data were extracted from digital hospital records and included demographic information, tumor characteristics, surgical reports, and histopathological findings, as detailed in the previous section.

Statistical analyses were conducted using IBM SPSS Statistics, version 29.0.0.0 (IBM Corp., Armonk, NY, USA). Recurrence-free survival was analyzed using Kaplan–Meier survival curves, and intergroup differences were assessed using the log-rank test and Cox regression analysis, with hazard ratios (HR) and corresponding 95% confidence intervals (CI) calculated. The influence of histopathological variables (e.g., subtype, invasion depth, tumor location) on resection margin status and recurrence was evaluated using univariate chi-squared tests. Associations between categorical variables were further examined using contingency tables and chi-squared analyses with adjusted residuals. A p-value < 0.05 was considered statistically significant. In the subanalysis of histological subtypes, cases with an unknown subtype were excluded from the calculations.

## Results

### Age and gender distribution

**Figure 1:**
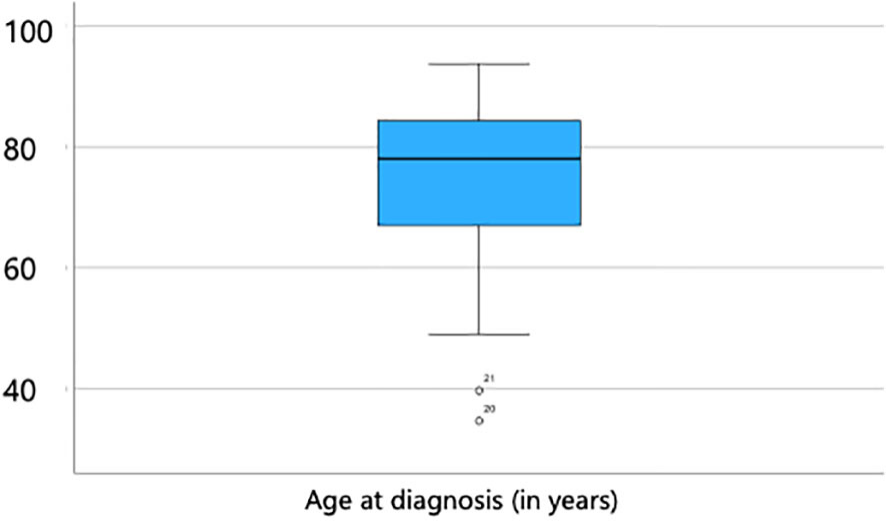
Age distribution at the time of diagnosis, ranging from 34.7 to 93.7 years, with a mean age of 75.4 years (SD = 11.1) and a median age of 78.1 years.

Among the 241 patients included in the study, 159 were male and 82 were female, resulting in a male-to-female ratio of approximately 2:1. The age at the time of diagnosis ranged from 34.7 to 93.7 years, with a mean age of 75.4 years (SD = 11.1) and a median age of 78.1 years (**Figure 1**). Neither patient age nor sex showed a significant association with incomplete (R1) resection status or tumor recurrence (p < 0.05).

### Tumor localization

**Figure 2:**
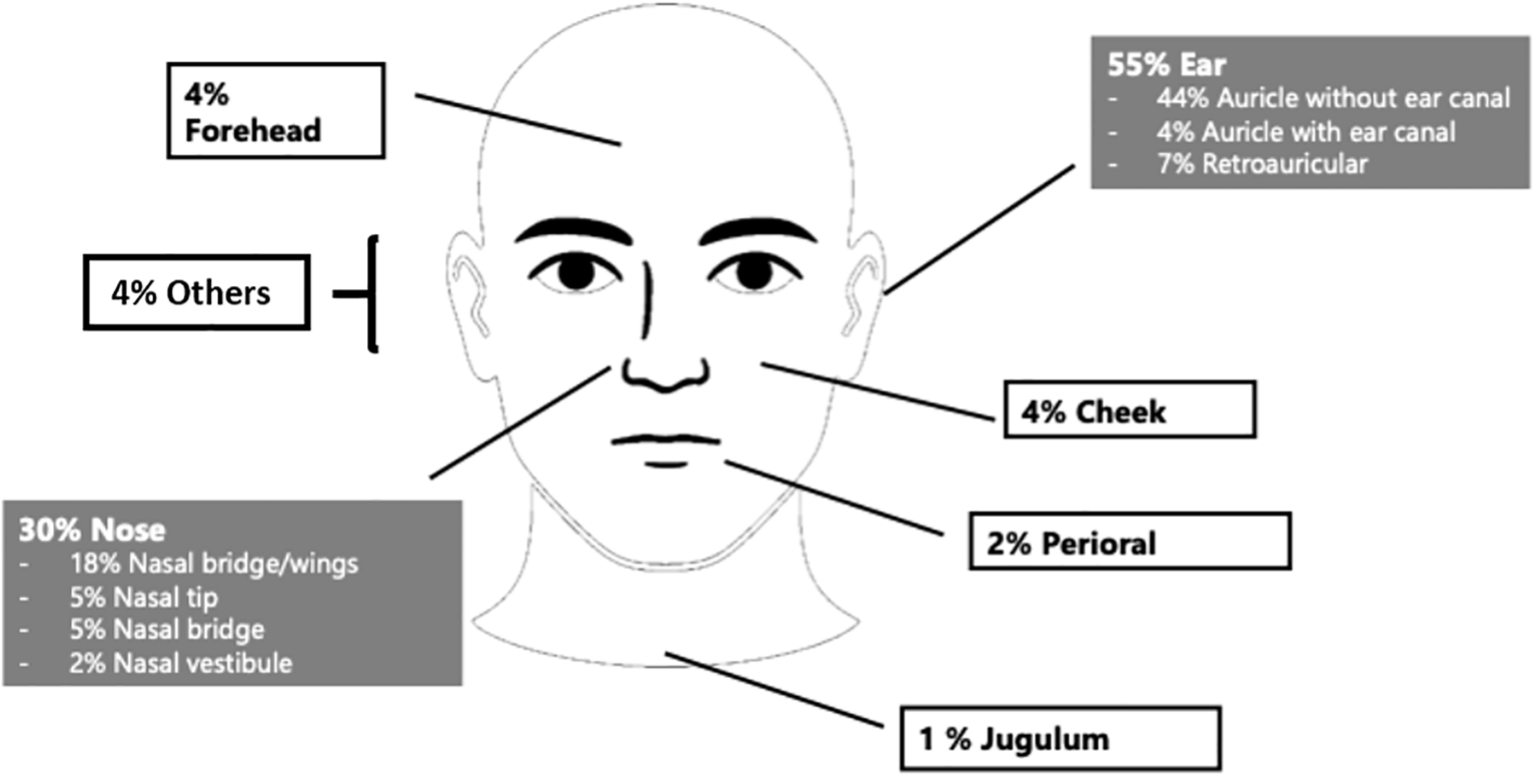
Tumor localization in the head and neck region: ear (55%), nasal (30%) and other facial sites (15%).

The majority of BCC were located in the ear region (n = 132; 54.8%) and nasal region (n = 71; 29.5%). Within the ear region, tumors were situated on the auricle with external auditory canal involvement in 9 cases (3.7%), on the auricle without canal involvement in 107 cases (44.4%), and in the retroauricular area in 16 cases (6.6%). Nasal tumors were distributed across the nasal tip (12 cases, 5.0%), nasal dorsum (11 cases, 4.6%), nasal sidewall/alae (44 cases, 18.3%), and nasal vestibule (4 cases, 1.7%). The remaining tumors (15.7%) were located in other facial areas, including the eyelid, forehead, cheek, periauricular region, lips, and jugulum (**Figure 2**).

### Histological subtypes

**Figure 3:**
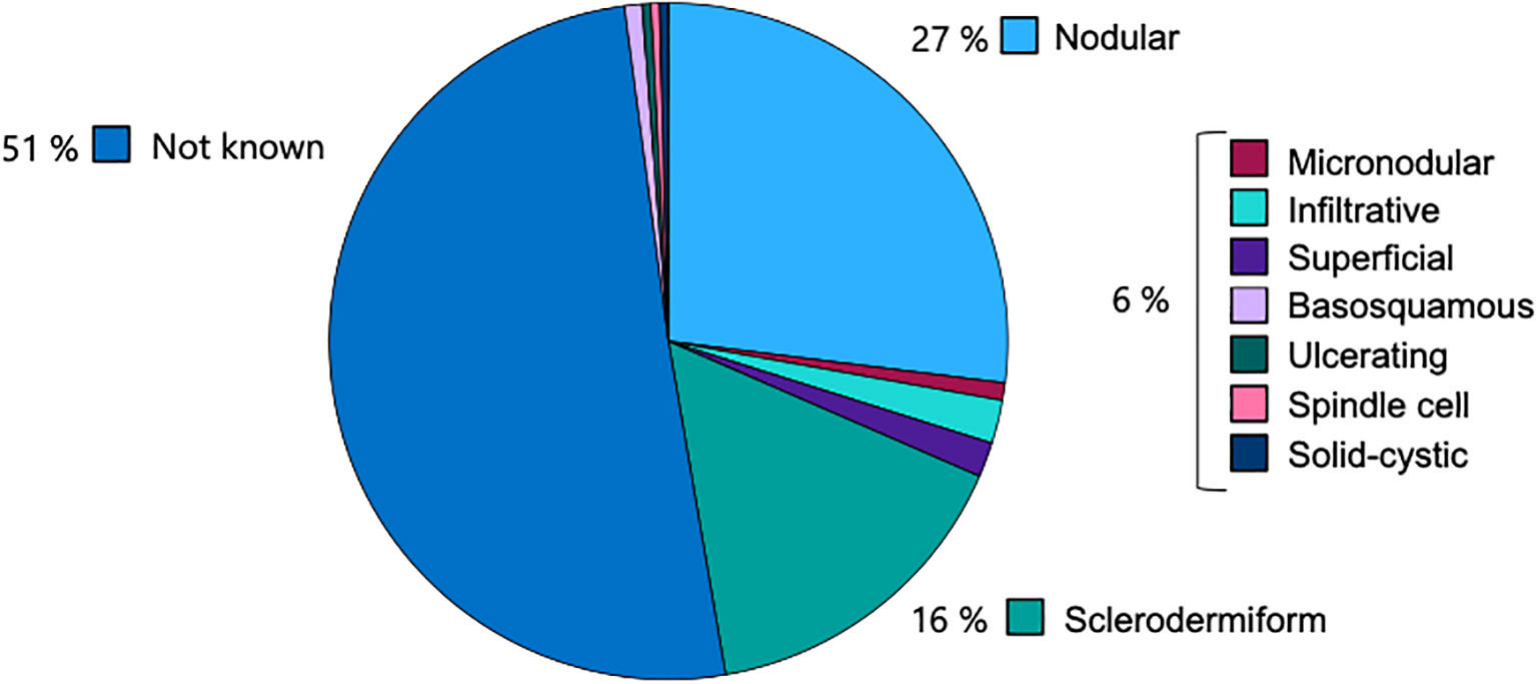
Distribution of histological subtypes of BCC, with nodular (27.0%) and sclerodermiform (15.8%) being most common among specified cases.

In 50.6% of cases, the histological subtype of BCC was not specified. Among the identified subtypes, nodular BCC was the most prevalent (27.0%), followed by the sclerodermiform subtype (15.8%). Less common subtypes included infiltrative (2.1%), superficial (1.7%), and micronodular (0.8%). Rare variants such as basosquamous (0.8%), ulcerating (0.4%), spindle cell (0.4%), and solid-cystic (0.4%) subtypes were also observed (**Figure 3**).

### Histopathological margin assessment and R0 resection rates in total

Histopathological margin assessments were performed externally in 27% of cases and internally in 73%. Referrals were primarily made by general practitioners, outpatient ENT specialists, and dermatologists.

Tissue sampling methods included biopsy (n = 111; 46%) and excisional biopsy (n = 130; 54%). Among the excisional biopsies, complete tumor resection (R0 status) was achieved in 52 cases (40%). All patients with non in sano resection underwent re-excision until R0 status was achieved, except for two noncompliant patients who declined further surgery. In the therapeutic surgical pathway (n = 241), the number of surgical stages required to achieve tumor clearance varied: 102 cases (42.3%) required one stage, 95 (39.4%) required two stages, 32 (13.3%) required three stages, 11 (4.6%) underwent four stages, and one case (0.4%) required five stages (**Table 1**).

**Table 1:** Overview of tissue sampling methods and distribution of the number of surgical stages required to achieve tumor clearance in the therapeutic pathway.

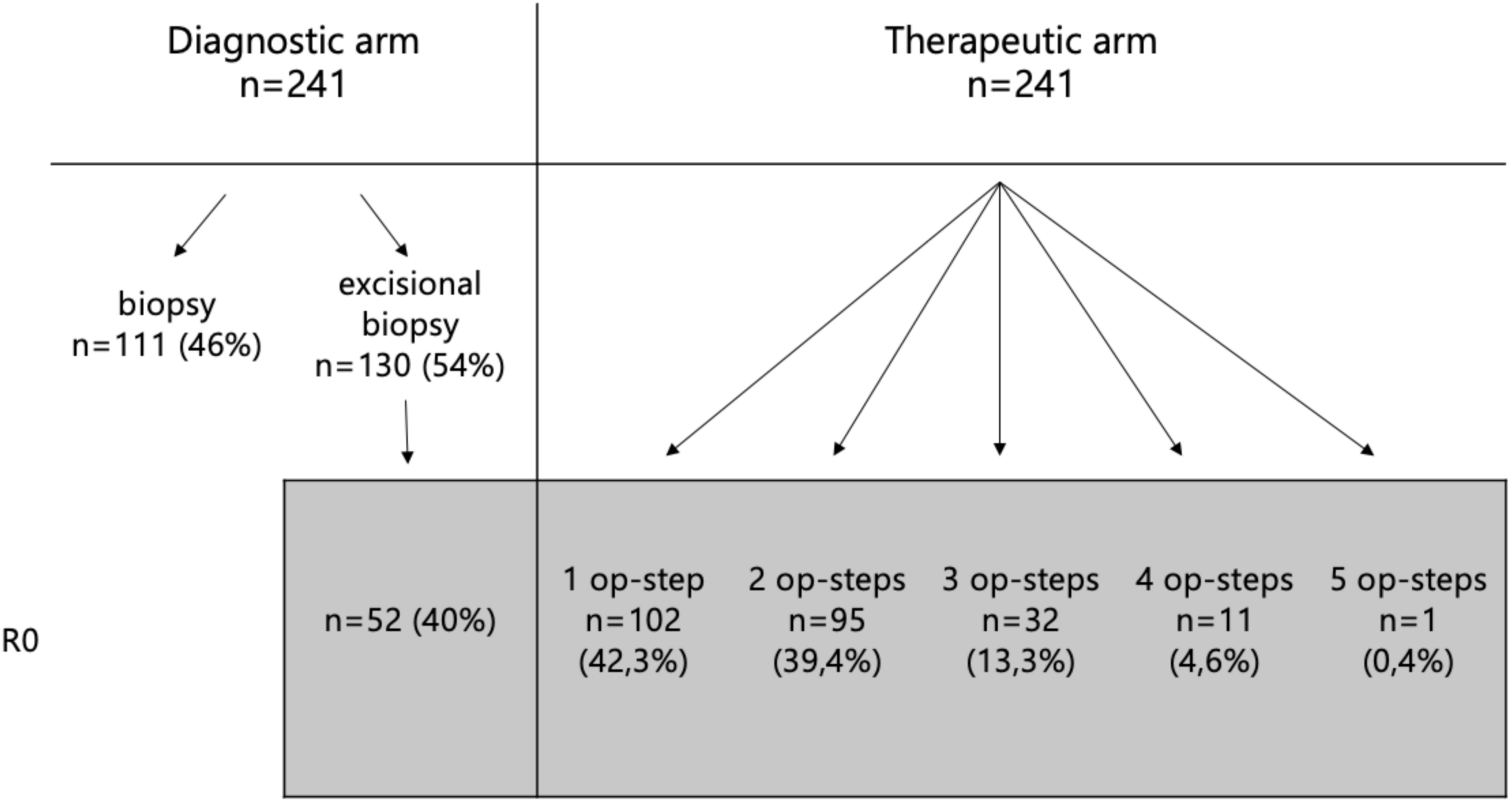

**Figure 4:**
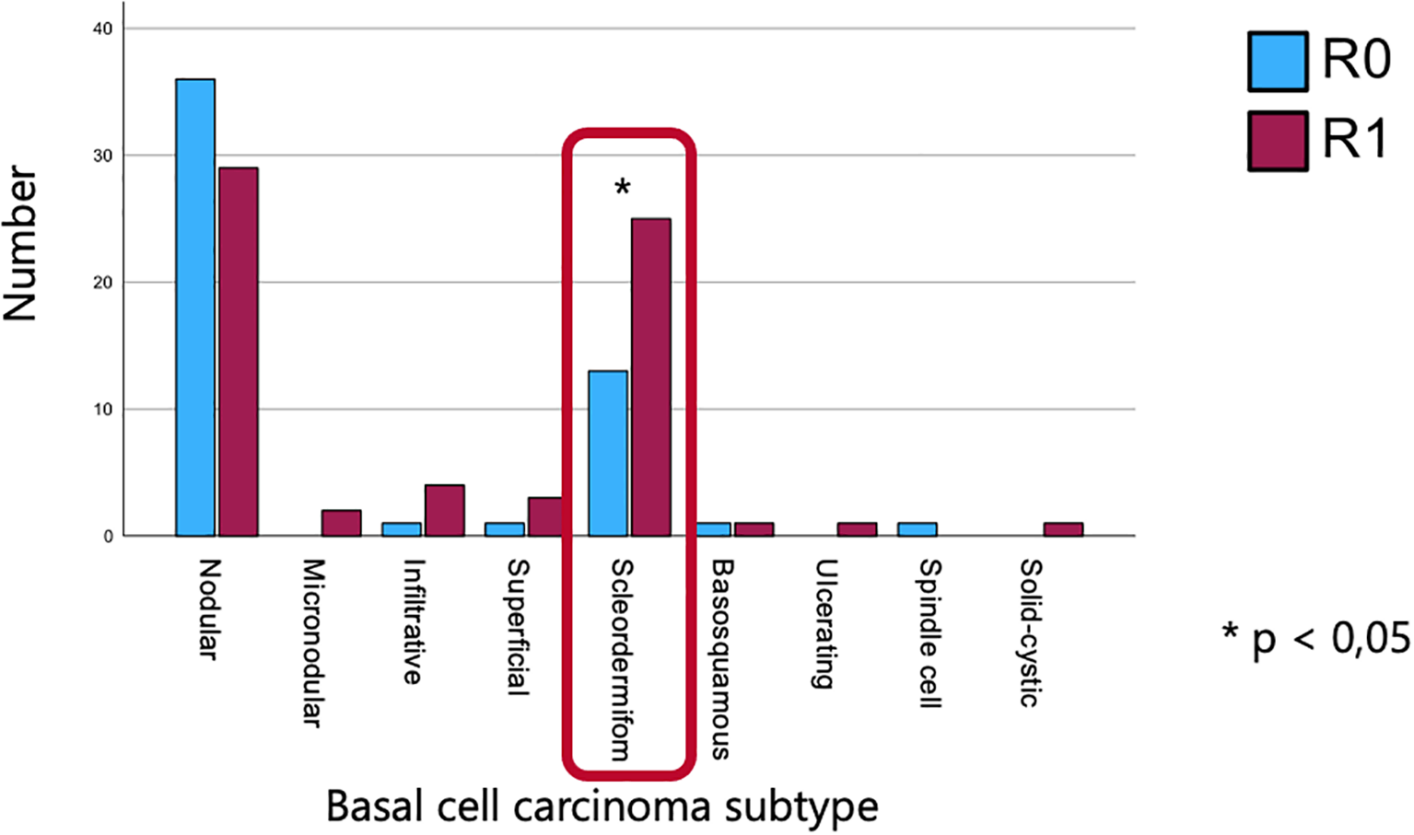
Association between histological subtype and resection status, highlighting a higher rate of incomplete resections in the sclerodermiform subtype and a significant correlation between subtype and margin status.

The sclerodermiform subtype was associated with a higher rate of incomplete (R1) resections, with 25 out of 38 cases exhibiting positive margins. Statistical analysis revealed a statistically significant correlation between histological subtype and resection status (p = 0.026) (**Figure 4**).

**Figure 5:**
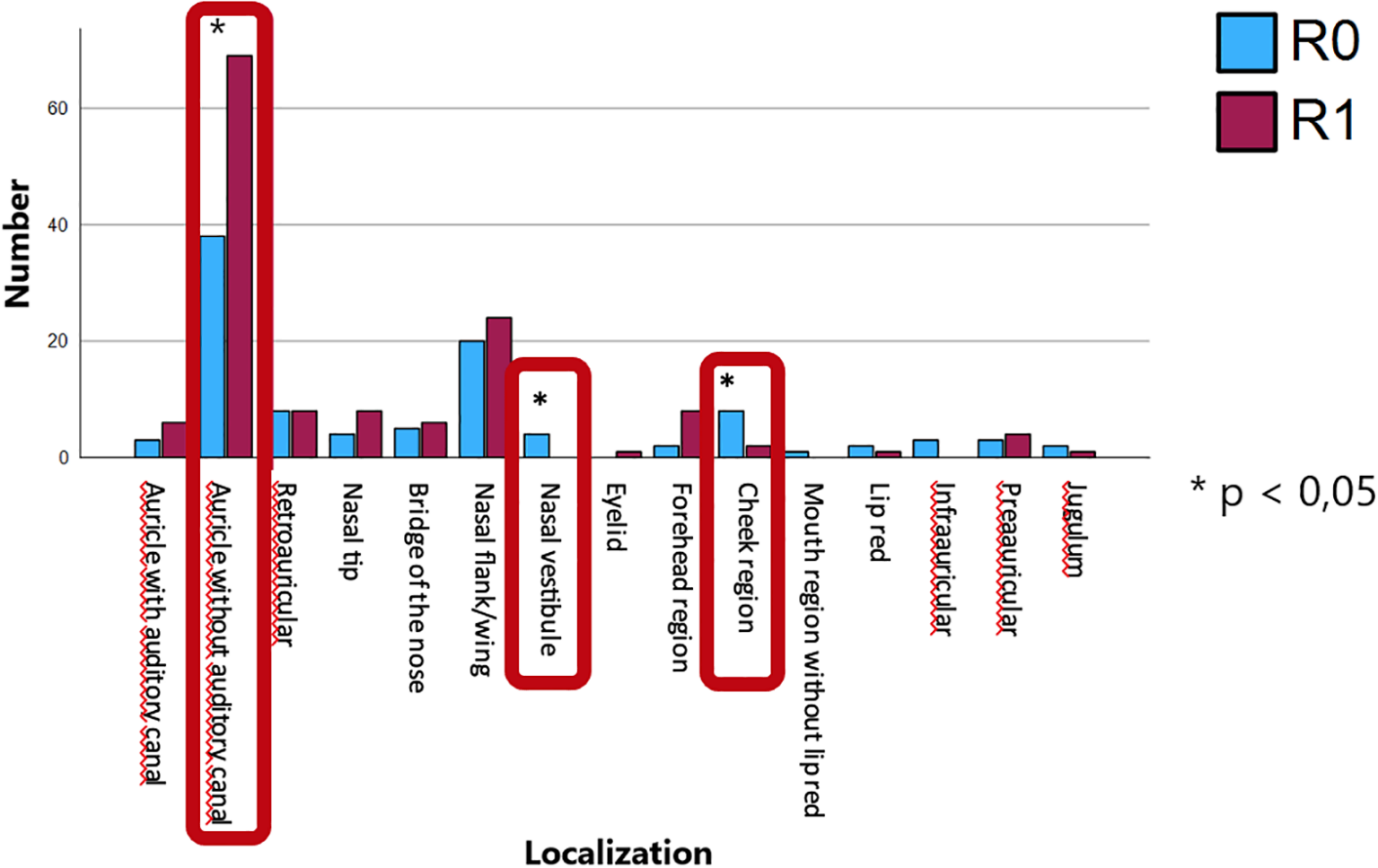
Anatomical localization and its association with R1 resection status, with lesions on the auricle (excluding the external auditory canal) showing a significantly higher rate of incomplete resections and lesions on the nasal vestibule and the cheek region showing a significantly higher rate of complete resections.

To investigate further predictors of R1 resection status, chi-squared analyses was conducted using tumor diameter (mean: 5.10 mm; SD: 1.44 mm), tumor depth (mean: 1.87 mm; SD: 1.61 mm), and anatomical localization as independent variables. Tumor diameter was not significantly associated with R1 status (p = 0.562) as well as tumor depth (p = 0.481) (**Table 2**). Anatomical localization was significantly associated with R1 status (p = 0.043), with lesions on the auricle (excluding the external auditory canal) demonstrating a higher rate of R1 resections and lesions on the nasal vestibule and the cheek region showing a significantly higher rate of complete resections (**Figure 5**).

**Table 2:** Tumor characteristics and adjusted residuals.

Tumor diameter in mm (p = 0.562)						
	1	2	3	4	5	>5
*R0*	5 (+1.10)	4 (+0.73)	11 (+0.85)	9 (+0.81)	8 (-0.14)	52 (-1.65)
*R1*	3 (-1.10)	3 (-0.73)	10 (-0.85)	8 (-0.81)	11 (+0.14)	80 (+1.65)

**Table:** 

Tumor depth in mm (p = 0.481)							
	<1	1	2	3	4	5	>5
*R0*	22 (-1.12)	21 (+0.43)	22 (-1.00)	17 (+0.10)	11 (+1.63)	3 (+0.78)	6 (+0.80)
*R1*	38 (+1.12)	25 (-0.43)	37 (+1.00)	22 (-0.10)	7 (-1.63)	2 (-0.78)	5 (-0.80)

**Table:** 

Localization (p = 0.043)								
	Auricle with auditory canal	Auricle without auditory canal	Retroauricular	Nasal tip	Bridge of the nose	Nasal flank wing	Nasal vesti-bule	Eyelid
*R0*	3 (-0.58)	38 **(-2.03)**	8 (+0.61)	4 (-0.68)	5 (+0.19)	20 (+0.40)	4 **(+2.33)**	0 (-0.87)
*R1*	6 (+0.58)	69 **(+2.03)**	8 (-0.61)	8 (+0.68)	6 (-0.19)	24 (-0.40)	0 **(-2.33)**	1 (+0.87)
	Forehead	Cheek region	Mouth region without lip red	Lip red	Infraauricular	Preauricular	Jugulum	
*R0*	2 (-1.48)	8 **(+2.43)**	1 (+1.16)	2 (+0.84)	3 (+2.02)	3 (+0.01)	2 (+0.84)	
*R1*	8 (+1.48)	2 **(-2.43)**	0 (-1.16)	1 (-0.84)	0 (-2.02)	4 (-0.01)	1 (-0.84)	

Adjusted residuals are shown in parentheses. Values > 2 indicate significant deviations (p < 0.05). Positive values indicate categories overrepresented in the group; negative values underrepresented.

### Subanalysis of excisional biopsis

In the subgroup of patients who underwent excisional biopsy (n = 130), histopathological margin assessment revealed no statistically significant association between resection status (R) and tumor depth (p = 0.132). Analysis of adjusted residuals indicated that cases without infiltration (<1 mm) occurred less frequently in the R0 group and more frequently in the R1 group than expected. However, no other depth categories showed significant deviations from the expected frequencies, suggesting a largely comparable distribution of tumor depth between the R0 and R1 groups.

Similarly, the distribution of tumor diameter in excisional biopsies did not differ significantly between R0 and R1 cases (p = 0.948). All adjusted residuals fell within the ±2 range, indicating no substantial deviation from expected values. Thus, tumor size at excisional biopsy appears to be comparable between complete (R0) and incomplete (R1) resections.

No statistically significant relationship was found between histologic BCC subtype and resection status in the excisional biopsy subgroup (p = 0.179). Adjusted residuals suggested a tendency for nodular subtypes to occur more frequently among R0 cases and for sclerodermiform subtypes to be more common in R1 resections, although these differences did not reach statistical significance.

Regarding tumor localization and resection margin status in excisional biopsy cases, adjusted residuals revealed that lesions located in the nasal vestibule and cheek occurred more frequently in R0 resections than expected, whereas tumors of the auricle were more common in R1 cases (p = 0.02). All other localization categories showed no substantial deviations from the expected frequencies.

### Type of anesthesia and organ preservation

Local anesthesia was used in 90.4% of procedures (n = 208), while general anesthesia was employed in 9.6% (n = 22). Organ preservation was achieved in 93.5% of cases (n = 225), whereas 6.5% (n = 15) required surgery without organ preservation. Among the non–organ-preserving procedures, 6 involved the nasal region and 9 involved the ear region. In 87% of non–organ-preserving cases, surgery had to be performed under general anesthesia.

### Recurrence and recurrence-free survival

Tumor recurrence was observed in five patients (2.1%) during the follow-up period. Kaplan–Meier and Cox regression analyses—including hazard ratios with 95% confidence intervals, all crossing 1 and therefore not statistically significant—revealed no significant association between recurrence or recurrence-free survival and any of the evaluated parameters, including tumor depth, histological subtype, anatomical location, or number of surgical stages. Recurrence-free survival was defined as the time from the date of initial histopathological diagnosis to either the date of histopathologically confirmed recurrence or, in the absence of recurrence, to the predefined censoring date of January 1, 2025.

### Wound coverage and local flap reconstruction

**Figure 6:**
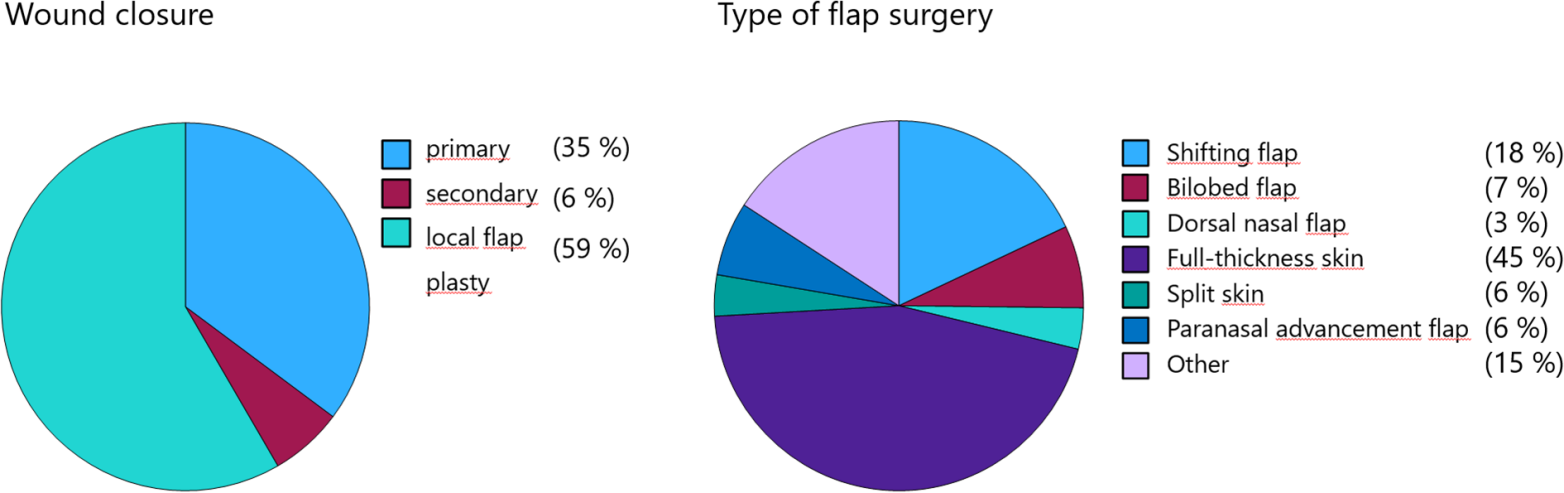
Reconstructive techniques following BCC excision, with local flaps and full-thickness skin grafts being most frequently used.

Primary wound closure was performed in 35.2% of cases, and secondary intention healing in 6.4%. The majority of defects (58.4%) were reconstructed using local flap techniques. Among these, full-thickness skin grafts were the most common method (45.3%), followed by advancement flaps (18.0%) and bilobed flaps (7.2%). Additional techniques included paranasal advancement flap (Nelaton) (6.5%), dorsal nasal flap (Rieger) (3.6%), and split-thickness skin grafts (3.6%). Other methods were used in 15.8% of cases (**Figure 6**). In 40% of cases, wound coverage was performed on the same day as the complete (R0) tumor resection, whereas 60% underwent a two-stage reconstruction. Immediate reconstruction was exclusively performed using advancement flaps and full-thickness skin grafts. No flap revision was required due to an R1 situation in cases treated with single-stage reconstruction.

## Discussion

This retrospective analysis of 241 cases of cutaneous BCC in the head and neck region provides valuable insights into patient management, surgical strategies, resection margin status, recurrence rates, and reconstructive approaches.

Our results confirm that BCC can be effectively managed under local anesthesia as an outpatient procedure in the majority of cases. This aligns with current literature emphasizing local anesthesia as the standard approach for most skin cancer treatments due to its safety, efficiency, and minimal systemic burden. Intracutaneous lidocaine is widely used and has a very low complication rate (1, 2).

Especially in elderly patients or those with well-defined lesions, local anesthesia facilitates outpatient treatment and supports organ preservation with excellent functional and cosmetic outcomes (3). In our cohort, over 90% of patients were treated under local anesthesia, and organ preservation was achieved in more than 93% of cases, even in sensitive areas such as the ear and nose. In the small subset of patients who required non–organ-preserving surgery, general anesthesia (ITN) was used in the majority of cases (87%). This was due to the greater complexity of these procedures, which often involved higher blood loss, more extensive tissue manipulation, increased postoperative pain, and more elaborate reconstructive requirements. These factors necessitate ITN to ensure patient safety and optimal surgical conditions.

Complete tumor resection (R0 status) was achieved in approximately 80% of cases with no more than two surgical stages. This confirms that high R0 resection rates for BCC can be achieved with minimal surgical staging. Our findings align with recent results by Iurilli et al. (4), who analyzed over 3000 BCCs and reported a 93.3% R0 rate using standard surgical techniques. A multicenter study by Ali et al. (5) further supports these findings, demonstrating that 6 mm margins yield a 95% histological clearance rate. Ozbey et al. demonstrated that the rate of positive surgical margins after surgery ranges between 9% and 37.2% (6).

Notably, 40% of excisional biopsies already resulted in complete resection, which is consistent with prior findings suggesting that excisional biopsy may suffice for small, well-defined BCCs (7). These results argue against more radical primary interventions and support tissue-preserving strategies, in line with current German S2k guidelines (8).

Analysis of resection margins in our cohort revealed significantly higher R1 resection rates in sclerodermiform BCCs and tumors located on the auricle and higher R0 rates in tumors located on the nasal vestibule and the cheek region. These findings are consistent with existing literature (9–11) identifying the sclerodermiform subtype as having indistinct clinical borders and aggressive, infiltrative growth patterns, which complicate complete excision. Reported R1 rates in sclerodermiform BCCs can reach up to 30%, compared to <10% in nodular variants (12, 13).

The auricle represents a particularly high-risk site for incomplete excision. Several studies (12, 14) have demonstrated that BCCs located at the ear are significantly more likely to result in R1 resections compared to other head and neck regions. A recent retrospective cohort study found that auricular BCCs (around 50 patients, 10.1% of the cohort) carry an odds ratio of 3.00 for incomplete excision when compared with tumors at other anatomical locations (13). Additional studies have confirmed that both the ear and the nose exhibit higher R1 rates, whereas sites such as the cheek present lower risk (15). These findings are attributed not only to the complex cartilage-rich anatomy of the auricle, which limits the ability to achieve adequate margins, but also to the increased prevalence of aggressive subtypes in this region (14, 16).

Given these anatomical and histopathological risk factors, early histologic subtype identification is crucial. In addition, careful surgical planning is required in auricular tumors to minimize the risk of incomplete resection and local recurrence.

Only 5 cases (2.1%) in our cohort experienced recurrence during follow-up, and no significant correlation was found between recurrence-free survival and parameters such as tumor depth, histologic subtype, tumor localization, or the number of surgical steps. These findings are consistent with previous reports indicating that a consistent strategy of re-resection in cases of positive margins can achieve excellent long-term tumor control.

In our study, we did not adopt a watch-and-wait approach for R1 resections. Instead, all patients were re-operated until complete (R0) excision was confirmed. The only exceptions were two patients who explicitly refused further surgery despite R1 status and were therefore considered noncompliant. This strict re-resection strategy likely contributed to the very low recurrence rate observed.

Multiple studies demonstrated that re-excision after incomplete (R1) resection significantly reduces recurrence rates compared to observation alone, particularly in high-risk anatomical sites and aggressive histologic subtypes (4, 17, 18). However, the low recurrence rate observed in our cohort contrasts with broader literature, which reports local recurrence rates for BCC in the head and neck region of approximately 10–15% over a 3- to 5-year period following standard surgical excision (19–21).

Reconstructive surgery favored local flap techniques in over 58% of cases, with full-thickness skin grafts (45%), advancement flaps (18%), and bilobed flaps (7%) being most common. This reflects the high priority given to aesthetic and functional outcomes in facial skin cancer surgery. Systematic reviews and large case series confirm that local flaps are the most common and preferred reconstructive method for nasal and auricular defects, offering reliable vascularity and lower rates of contour irregularity or alar notching (22–24). Bilobed flaps are effective for the nasal ala and dorsum, with high rates of patient satisfaction and low complication rates (25).

Limitations of this study of this study include its retrospective design and a relatively short follow-up period of up to six years, which may limit the ability to detect late recurrences. However, a major strength of this study lies in the fact that all patients were followed up in a dedicated specialist consultation by the surgical team itself. This ensured standardized postoperative evaluation, minimized interobserver variability, and increased the reliability of recurrence assessments. Another limitation of this study is that cases with unknown histological subtype were excluded from the subgroup analysis, which may have introduced selection bias.

In conclusion, this study demonstrates that a structured, tissue-preserving surgical approach under local anesthesia is highly effective for the management of BCC in the head and neck region. High rates of complete tumor resection (R0) and organ preservation were achieved with minimal surgical staging. Incomplete excisions were primarily associated with the sclerodermiform subtype and auricular localization, underscoring the importance of preoperative risk stratification. The low recurrence rate observed—even in this anatomically complex region—supports consistent re-excision strategies in cases of positive margins. The frequent use of local flap and graft techniques reflects a strong emphasis on both oncologic safety and optimal aesthetic-functional outcomes.

Given the challenges in achieving complete tumor excision, particularly in sclerodermiform BCCs and auricular lesions, the integration of high-frequency ultrasound (HFUS, 20–50 MHz) and ultra-high-frequency ultrasound (UHFUS) into preoperative workflows appears highly promising. These non-invasive imaging techniques enable real-time assessment of tumor depth and subclinical extension, allowing for precise delineation of lateral and deep margins in high-risk and anatomically complex areas (26, 27). Early data suggest that ultrasound-guided surgical planning may reduce incomplete excision rates, facilitate more conservative resections, and improve both oncologic control and cosmetic outcomes. While not yet part of routine clinical protocols, current evidence supports the future role of HFUS and UHFUS in individualized treatment strategies for head and neck BCCs (28, 29).

## Data Availability

The raw data supporting the conclusions of this article will be made available by the authors, without undue reservation.
